# How cognitive psychology changed the face of medical education research

**DOI:** 10.1007/s10459-020-10011-0

**Published:** 2020-11-26

**Authors:** Henk G. Schmidt, Silvia Mamede

**Affiliations:** Department of Psychology, Erasmus University, P.O. Box 1738, 3000, DR Rotterdam, the Netherlands

**Keywords:** Knowledge acquisition, Self-explanation, Elaborative discussion, Distributed practice, Cognitive load, Retrieval practice, Interleaving practice, Medical expertise

## Abstract

In this article, the contributions of cognitive psychology to research and development of medical education are assessed. The cognitive psychology of learning consists of activation of prior knowledge while processing new information and elaboration on the resulting new knowledge to facilitate storing in long-term memory. This process is limited by the size of working memory. Six interventions based on cognitive theory that facilitate learning and expertise development are discussed: (1) Fostering self-explanation, (2) elaborative discussion, and (3) distributed practice; (4) help with decreasing cognitive load, (5) promoting retrieval practice, and (6) supporting interleaving practice. These interventions contribute in different measure to various instructional methods in use in medical education: problem-based learning, team-based learning, worked examples, mixed practice, serial-cue presentation, and deliberate reflection. The article concludes that systematic research into the applicability of these ideas to the practice of medical education presently is limited and should be intensified.

## Introduction

Research into medical education began to attract serious attention with the publication of the *Journal of Medical Education* (now *Academic Medicine*) in 1951. Not surprisingly, from its very beginning it has been influenced by what was current in the psychology of learning and instruction and always reflected its ongoing concerns. In the fifties and sixties the language of behaviorism was dominant in the medical education literature. Learning was seen as the result of repetition and reward, with its application to so called ‘learning machines’ (Owen et al. 1965, 1964), to programmed instruction (Lysaught et al. 1964; Weiss and Green 1962), and with its emphasis on ‘behavioral’ objectives (Varagunam 1971). Cognitive-psychology concepts such as ‘memory,’ ‘retention,’ and ‘reasoning’ started to appear only in the early seventies (Elstein et al. 1972; Klachko and Reid 1975; Levine and Forman 1973), and found an early synthesis in the groundbreaking work of Elstein and colleagues on medical problem solving (Elstein et al. 1978). The purpose of the present article is to assess the role of cognitive psychology in the study of medical education (and by extension health professions education). We will focus here on how cognitive conceptualizations of learning and instruction have assisted in an understanding of knowledge acquisition and expertise development in medicine. Of course, these two topics, knowledge acquisition and expertise development, are closely intertwined. However, the study of clinical reasoning is so vital to medical education and has seized upon its own niche within the research community, that we will discuss it separately. Since this article was written to contribute to the celebration of the 25th anniversary of *Advances in Health Sciences Education*, references are to articles published by this journal whenever possible. First however we present a crash course in the cognitive psychology of knowledge acquisition.

## A brief introduction to the cognitive psychology of knowledge acquisition

When first-year medical students are confronted with information new to them from a chapter of Guyton and Hall’s textbook of medical physiology, they *activate prior knowledge* from high-school or college biology to help them interpret the new information; they use existing knowledge to *construct* new knowledge. This new understanding, if sufficient thorough, is *stored in long-term memory* to be used for subsequent learning or application (Anderson et al. 2017). What can be learned however is also dependent on *limitations of working memory*, the part of memory where knowledge is consciously processed (Baddeley and Hitch 1974; Mayer 2010). Finally, knowledge needs to be biologically *consolidated* in memory in order to survive (Lee 2008; McGaugh 2000). This consolidation is biochemical in nature first, then synaptic. These processes take several hours to stabilize. It is well-known that memory for things learned is much better after a good night sleep. A third and final process is systems consolidation in which memories are moved from the hippocampal area to the cortex and become indestructible—although not necessarily retrievable (Winocur and Moscovitch 2011). This process takes years. Retrievability is influenced by the extent to which students apply their knowledge in contexts of sufficient variability and the extent to which these contexts resemble the *context* in which it was learned initially (Eva et al. 1998; Norman 2009).

## Instructional interventions that foster learning

The cognitive processes described above, delineating what the mind, engaged in learning, does naturally, can be boosted by instructional interventions. We will first describe these interventions here, focusing on the most important ones. Some of these interventions aim at strengthening the relationship between prior knowledge and new information. Others attempt to facilitate processing of information. A third category aims to strengthen long-term memory. In a subsequent section we will relate these interventions to some of the most prevalent instructional approaches to medical education developed since the early seventies.

### Interventions aimed at strengthening the relationship with prior knowledge

#### Encouraging self-explanation

Self-explanation is a form of *elaboration* upon what is learned. The students do this by relating new information to knowledge previously acquired or repeat the information verbally in their own words (Chi et al. 1989, 1994). Elaboration is known to be more helpful than simple repetition of new material (Craik and Lockhart 1972). Chi et al. (1994) found that students who were asked to self-explain after reading each line of a passage on the human circulatory system had a significantly greater knowledge gain from pre- to posttest than students who read the text twice. In an experiment of van Blankenstein et al. (2011) students either listened to an explanation provided for a particular problem or had to generate an explanation themselves, before studying an appropriate text. There were no immediate effects on retention of the text. However, one month later, participants who had actively engaged in self-explanation remembered 25% more from the text.

#### Facilitating elaborative discussion

If students are allowed to discuss subject matter with peers or are being prompted by a teacher, learning improves considerably. In a meta-analysis of small-group learning in science, mathematics, engineering, and technology (Springer et al. 1999) found effects on learning considerably more sizable than those of most other educational interventions. Versteeg et al. (2019) studied how elaborative discussion among peers would foster understanding of physiology concepts compared with individual self-explanation and a control condition. They found that the elaborative-discussion group outperformed the self-explanation group, while both outperformed the control group. Interestingly, students with initially wrong concepts profited even when discussing them with a peer who also had an initial wrong understanding.

#### Promoting distributed practice

If one spreads learning and retrieval activities over time, returning to the same contents a couple of times, knowledge become better consolidated. Distributed-study opportunities usually produce better memory than massed-study opportunities (Delaney et al. 2010). It turned out difficult however to find a suitable example of the effects of massed versus spaced practice in medical education. Kerfoot et al. (2007) conducted a number of studies in which they sent to residents at regular intervals emails on four urology topics. These emails consisted of a short clinically relevant question or clinical case scenario in multiple-choice question format, followed by the answer, teaching point summary, and explanations of the answers. Students were randomized to receive weekly e-mailed case scenarios in only 2 of the 4 urology topics. At the end of the academic year, residents outperformed their peers on the questions related to the emails they had received. However, this effect could also be explained by mere exposure since the residents apparently had not received the same information in massed form.

### Interventions aimed at facilitating processing of new information

#### Help in decreasing cognitive load

As indicated above, working memory allows for only limited information to be processed at the same time. If the cognitive load of information exceeds what can be processed, learning is hampered (van Merrienboer and Sweller 2010). Much research has gone into the question how cognitive load could be optimized by instruction. One successful strategy is the use of worked examples. Rather than require students to solve problems in a particular domain by themselves, the teacher presents worked-out examples of these problems for study (Chen et al. 2015). The assumption here is that by seeing all elements required to solve a problem, decreases cognitive load. Students with limited knowledge seem to profit from such approach, whereas students with enough knowledge are sometimes hampered (Kalyuga et al. 2001).

### Interventions aimed at strengthening long-term memory

#### Fostering retrieval practice

When you ask students to retrieve information previously learned from memory, for instance by providing them with regular quizzes, knowledge reactivated this way becomes more entrenched in memory. Dobson and Linderholm (2015) for instance, had students reading anatomy and physiology texts either three times, two times with the possibility of making notes, or two times interspersed by an attempt to retrieve as much information as possible. After a one-week retention interval, those who engaged in retrieval practice demonstrated superior performance compared to the other two groups.

#### Fostering interleaving practice

Offering cases with different diagnoses in a clinical reasoning exercise boosts learning because students learning to distinguish between cases that look the same but have different diagnoses, and cases that look different but have the same diagnosis. Interleaving may slow initial learning but, in the end, leads to better retention and application. An illustrative example is provided by Hatala et al. (2003). They presented students with electrocardiograms with the aim to learning to diagnose such ECGs. In one of their experiments, students were randomly allocated to one of two practice phases, either "contrastive" where examples from various categories are mixed together, or "non-contrastive" where all the examples in a single category are practiced in a single block. Students in the mixed-examples condition outperformed those in the blocked-practice condition while diagnosing a set of new ECGs. See for another example Kulasegaram et al. (2015).

## To what extent are these interventions applied to the practice of medical education?

No doubt, these interventions are sometimes applied by teachers in their courses on an individual basis. Teachers allow students to discuss subject matter in small groups or provide quizzes during their lectures. However, there have been attempts, most of them only during the last twenty years, to develop instructional models explicitly based on cognitive principles as discussed above. We will outline four of these: Problem-based learning, team-based learning, worked examples, and mixed practice.

*Problem-based learning.* (PBL) was actually an early innovation. It was developed at McMaster University, Canada where in 1969 a first group of 20 students entered medical school. PBL has the following six defining characteristics: (i) Biomedical or clinical problems are used as a starting point for learning; (ii) students collaborate in small groups for part of the time; (iii) under the flexible guidance of a tutor. Because problems are the trigger for learning (iv) the curriculum includes only a limited number of lectures; (v) learning is student-initiated, and (vi) the curriculum includes ample time for self-study. For the founding staff PBL was merely a combination of good educational practices aimed at increasing motivation among students (Servant-Miklos 2019a). However, by the end of the seventies, and due to work done at Maastricht University, the Netherlands, PBL underwent a reinterpretation in line with cognitive psychology findings (Schmidt 1983; Servant-Miklos 2019b). Table 1 contains the authors’ labelling of cognitive processes and interventions underlying PBL (Schmidt et al. 2011).

**Table 1:** Extent to which cognitive principles are actualized in four instructional models

	Problem-based learning	Team-based learning	Worked examples	Mixed practice
Activation of prior knowledge	+ +	+ +	+	+
Consolidation	−	+ +	−	−
Appropriate context	+ +	+	+ +	+ +
Self-explanation	+ +	+ +	−	−
Elaborative discussion	+ +	+ +	−	−
Decreasing cognitive load	−	−	+ +	−
Retrieval practice	+	+ +	−	−
Distributed practice	−	+	−	+ +
Interleaving practice	−	−	−	+ +

+  + means that according to literature the principle is explicitly operationalized in the instructional model. + means that it can be expected to play a role although not explicitly assumed.—means that it does not play a role

*Team-based learning* (TBL) was developed in 1997 by Larry Michaelsen at the University of Central Missouri, US, when increasing class sizes prevented him from teaching in the Socratic fashion (Michaelsen et al. 2002). The idea emerged for the first time in the medical education literature in 2005 (Koles et al. 2005). TBL consists of three phases: (i) A preparatory phase, in which students study individually preassigned materials often conveyed through video; (ii) an in-class readiness assurance phase, consisting of an individual test, a subsequent retest taken after discussion of the answers to the individual test are discussed in a team, and teacher feedback; (iii) an in-class application phase in which students through facilitated interteam discussion solve new problems and answer new questions derived from the initial learning materials. Schmidt et al. (2019) and colleagues have recently provided the cognitive account of what happens to the learner in TBL as outlined in Table 1.

**Table 2:** Numbers of studies published in Advances in Health Sciences Education between 1995 and 2020 applying cognitive principles and instructional models

Cognitive principles	No of articles	Instructional models	No of articles
Activation of prior knowledge	29	Problem-based learning	121
Consolidation	2	Team-based learning	4
Appropriate context	16	Worked examples	3
Self-explanation	7	Mixed practice	4
Elaborative discussion	21	Teaching of clinical reasoning	17
Decreasing cognitive load	17		
Retrieval practice	4		
Distributed practice	0		
Clinical reasoning	62		

*Worked examples* are common in text books on physics, mathematics and chemistry. It was probably Sweller and Cooper (1985) who saw their potential for reducing cognitive load while problem solving. In the previous section we have already provided a successful example of the application of cognitive load theory in the health professions field (Chen et al. 2015). However, the number of studies on worked examples reported in that literature is still limited. A search into the three most-cited journals in health professions education, Academic Medicine, Medical Education, and Advances in Health Sciences Education unearthed 15 articles, the oldest being from 2002. The use of worked examples would potentially be a fruitful addition to the arsenal of methods used to teach clinical reasoning, but we definitively need more studies.

*Mixed practice* or interleaving has large potential for medical education, in particular because one of its important functions is the teaching of diagnostic problem solving (Richland et al. 2005; Rohrer 2012). Cases that superficially look the same may have different causes. Alternatively, cases demonstrating a quite different array of symptoms, may have the same underlying pathology. Training student to compare and contrast such cases would be optimal using this instructional approach. However, only six illustrative examples could be found in the extant health professions literature, interestingly most of them provided by Geoffrey Norman, and his associates from McMaster University.

Table 1 summarizes the extent to which each of the cognitive principle discussed in the previous section are actualized in these four instructional approaches.

## The study of medical expertise

Medical expertise is an attractive domain of study for cognitive psychologists. This is so not only because the quality of our care as patients depends on the performance of our physicians but also because of peculiar features of the medical practice. Physicians operate upon an extremely broad and complex knowledge basis, and clinical problem-solving involves a large spectrum of cognitive processes, ranging from attention and perception to decision-making. Not surprisingly, medical expertise has drawn researchers’ attention over four decades (Norman 2005). This research has focused on clinical reasoning, particularly the diagnostic process. One of major goals of medical education is to develop students’ clinical reasoning and helping students become good diagnosticians is much valued. Medical expertise research has contributed substantially to our understanding of how this goal can be achieved (or at least how it should be pursued). The following session summarizes the main contributions of this research to what we know about, first, the nature of clinical reasoning and, second, how it develops in medical students. Subsequently, we will discuss the impact of this research on medical education, particularly how its contributions have interacted with conceptualizations of learning and instruction discussed earlier in this article to inform the teaching of clinical reasoning.

## The nature of clinical reasoning

The major findings that have shed light on the nature of clinical reasoning can be grouped into three subheadings that parallels the history of the research on the subject.

### The ‘hypothetico-deductive’ method as a general model of clinical problem-solving

Early in a clinical encounter, physicians generate one or a few diagnostic hypotheses and subsequently gather additional information to either confirm or refute these hypotheses. This ‘hypothetico-deductive’ method was revealed by pioneering studies conducted in the 1970s using traditional methods of cognitive psychology research, such as observing physicians and students interacting with standardized patients while thinking aloud (Elstein et al. 1978, 2009). These studies attempted to uncover the reasoning process that characterizes experts’ reasoning, which could then be taught to students. However, although the hypothetico-deductive method provides a general representation of diagnostic reasoning, subsequent studies soon showed that it does not explain expert performance (Elstein et al. 1978; Neufeld et al. 1981). Medical students also employed the same approach, and what differentiated expert and novice diagnosticians was not a particular reasoning process but rather the quality of their diagnostic hypotheses (Barrows et al. 1982). An additional crucial finding of the same period was that diagnostic performance on one clinical case did not predict performance on another case. The phenomenon, labeled by Elstein ‘content specificity’ (Elstein et al. 1978), was proved to happen even when the cases were within the same specialty (Eva et al. 1998; Norman et al. 1985).

### How medical knowledge is structured in memory and used in diagnostic reasoning

It is not a particular *process* that determines expert performance, but rather the *content* of reasoning, i.e. knowledge itself (Norman 2005). This conclusion came from a new era of studies conducted when researchers, faced with the aforementioned findings, turned attention to the kinds of medical knowledge, how knowledge is structured in memory and used to diagnose clinical problems. These studies relied heavily on methods from cognitive psychology research to carefully search from differences in knowledge structures of expert and non-expert diagnosticians. For example, many of these studies requested medical students at different years of training and (more or less) experienced physicians to diagnose clinical cases and subsequently explain the patient’s signs and symptoms or, alternatively, to solve the case while thinking-aloud. The resulting protocols were analyzed to identify the kinds and amount of knowledge used during diagnostic reasoning (Patel and Groen 1986; Schmidt et al. 1990). Several knowledge structures have been proposed, suggesting that diseases would be represented in memory, for example, as prototypes (Bordage and Zacks 1984), or as instances of previously seen patients (Norman et al. 2007), or yet as schemas and scripts (Schmidt et al. 1990). Some of these proposals, such as prototype models, consisted of application of representation models long existing in psychology to medical knowledge. Other authors however developed formats specifically for representing medical knowledge, such as the concept of illness scripts. Illness scripts are mental scenarios of the conditions under which a disease emerges, the disease process itself, and its consequences in terms of possible signs, symptoms, and management alternatives (Feltovich and Barrows 1984). Some empirical support exists for several proposals, and it is likely that (some of) these different knowledge structures coexist in physicians’ memory to be mobilized when needed (Custers et al. 1996; Schmidt and Rikers 2007).

These conceptualizations have framed our understanding of diagnostic reasoning. Notice that, despite their differences, they share the basic idea that diseases are associated in memory with a set of observable clinical manifestations. Briefly, the presence of some of these manifestations in a patient activates in the physician’s memory the mental representation of the disease, generating a diagnostic hypothesis. Search for additional information follows to verify whether other manifestations associated with the disease are actually present. When this search reveals findings that contradict the initial diagnosis and rather suggest others, new hypotheses may be activated and tested against the patient findings.

### The dual nature of diagnostic reasoning

Dual-process theories of reasoning, long studied in psychology, represent another approach to understanding and conceptualizing diagnostic reasoning. They assume that two different forms of reasoning exist, one that is associative, based on pattern-recognition, fast, effortless and largely unconscious (usually named System 1 or Type 1) and another that depends on applying rules, is slow, effortful and takes place under conscious control (System 2 or Type 2) (Evans 2008, 2006; Kahneman 2003). While Type 1 processes accounts for intuitive judgments, Type 2 processes have to take place when these judgments are verified. Appling this model to medical diagnosis, Type 1 reasoning would explain the generation of diagnostic hypotheses whose subsequent verification depends on Type 2 processes. Indeed, studies within the medical expertise research tradition seem in line with dual-process models. There is substantial evidence that physicians use non-analytical reasoning to arrive at diagnoses (Norman and Brooks 1997). Radiologists, for example, were able to detect abnormalities in medical images with around 70% accuracy in 200 ms (Evans et al. 2013; Kundel and Nodine 1975). Studies on the role of similarity in diagnosis also provide additional evidence: diagnostic accuracy increased when a dermatological case was preceded by a similar one (Brooks et al. 1991), and similarity affected the diagnosis even when what was similar in two cases was a diagnostically irrelevant feature (e.g. the patient occupation) (Hatala et al. 1999). There is also substantial evidence that physicians adopt both intuitive and analytical reasoning modes in different degrees depending on the circumstances such as the level of complexity of the case or perception of how problematic a case might be (Mamede et al. 2007, 2008).

Dual-process representations of diagnostic reasoning have become prominent in the medical literature (Croskerry 2009). A research tradition has grown triggered by increasing concerns with the problem of diagnostic error. Flaws in the physician’s cognitive processes have been detected in the majority of diagnostic errors (Graber 2005), and the sources of cognitive errors have been much discussed in the medical literature (Norman 2009; Norman et al. 2017). Several authors have attributed flaws in reasoning, and consequently errors, to cognitive biases induced by heuristics, shortcuts in reasoning frequent in Type 1 processes (Croskerry 2009; Redelmeier 2005). Conversely, other authors argue that heuristics are usually efficient and point to specific knowledge deficits rather than particular reasoning processes as the explanation for reasoning flaws (Eva and Norman 2005; McLaughlin et al. 2014; Norman et al. 2017). This controversy should not be seen as a theoretical discussion only, because it has direct consequences for medical education. While the first position demands educational interventions aimed at increasing trainees’ and practicing physicians’ ability to recognize biases and counteracting them, the second points to interventions that enhance knowledge acquisition and restructuring. We will return to this point when discussing the teaching of clinical reasoning. To discuss teaching, we need first to understand how clinical reasoning develops in medical students.

## The development of clinical reasoning in medical students

In the course towards becoming an expert, medical students move through different stages characterized by qualitatively different knowledge structures that underlie their performance (Schmidt et al. 1990; Schmidt and Rikers 2007). This *restructuring* theory of medical expertise development has come out of a research program focused on understanding how knowledge was organized in memory and used to solve clinical problems as students progress through education. In the first years of their training, students rapidly develop mental structures representing *causal networks* that explain the origins and consequences of diseases on the basis of their pathophysiological mechanisms (Schmidt et al. 1990; Schmidt and Rikers 2007). Studies that asked students at this stage to diagnose clinical problems showed that, because students still do not recognize patterns of connected symptoms, they try to explain isolated symptoms based on their causal mechanisms. This processing is effortful and detailed, with much use of basic sciences knowledge. This translated, for example, in the finding that students recalled more from a case than experts, which has become known as the ‘intermediate effect’ (Schmidt and Boshuizen 1993).

A first qualitative shift in knowledge structure occurs when students start to apply the knowledge that they have acquired to solve clinical problems. Gradually, the detailed knowledge of the chain of events that leads to a symptom is ‘encapsulated’ in more generic explanatory models or diagnostic labels that stands for the detailed explanation (Schmidt et al. 1990; Schmidt and Rikers 2007). Through this process, a small number of abstract, higher-order concepts, representing for example a syndrome or a simplified causal mechanism, ‘summarize’ a larger number of lower-levels concepts. For example, when students were requested to explain the clinical manifestations in a patient presenting with bacterial endocarditis and sepsis, they reasoned step-by-step through the chain of events that starts with the use of contaminated syringes until their consequences, i.e. the symptoms. Conversely, experts used the concept of ‘sepsis’ as a label that ‘encapsulates’ much of the chain of events, without the need to use this knowledge in their diagnostic reasoning (Schmidt et al. 1988). Many studies have shown experts to make much use of this type of ‘encapsulated’ concepts when reasoning through a case, leading to think aloud or recall protocols that contain less reference to basic sciences concepts or underlying mechanisms than the students’ ones (Boshuizen and Schmidt 1992; Rikers et al. 2004, 2000). However, basic sciences knowledge remains available and is indeed ‘unconsciously’ used during the diagnosis as studies with indirect measures of reasoning have shown (Schmidt and Rikers 2007).

A second shift in knowledge structures occurs as exposure to patients increases. Encapsulated knowledge is gradually reorganized into narrative structures that ‘represent’ a patient with a particular disease (Feltovich and Barrows 1984; Schmidt et al. 1990). These ‘illness scripts’ contain little knowledge of the causal mechanisms of the disease, because of encapsulation, but are rich in clinical knowledge about the enabling conditions of the disease and its clinical manifestations (Custers et al. 1998). Knowledge of enabling conditions tends to increase with experience and play a crucial role in expert physicians’ reasoning (Hobus et al. 1987). As exposure to actual patients increases, traces of previously seen patients are also stored in memory. Illness scripts exist therefore at different levels of generality, ranging from representations of disease prototypes to representations of previously seen patients (Schmidt and Rikers 2007).

Successful diagnostic reasoning seems to depend critically on developing rich, coherent mental representations of diseases (Cheung et al. 2018). For instance, a series of studies attempting to investigating the role of biomedical knowledge in diagnostic reasoning had students learning the clinical features associated with a disease either together with explanations of how they are produced or without explanation (Woods et al. 2007). Learning how the clinical features are connected by causal mechanisms led to higher diagnostic accuracy when diagnosing cases of the disease after a delay. Besides bringing additional evidence of the knowledge encapsulation process, these studies suggest that understanding their underlying mechanisms help ‘glue’ the clinical features together, leading to more coherent and stable mental representations of the diseases, which make it easier to recognize them when diagnosing similar cases in the future.

This body of research contributed to our understanding of how students develop the ability to diagnose clinical problems in the course of medical education and to set a for the design of interventions for the teaching of clinical reasoning.

## The teaching of clinical reasoning

The research described above provides substantial evidence that expert physicians do not employ any peculiar reasoning mode and there is no such thing as general reasoning skills that can be taught to students. Nevertheless, proposals for teaching students how to reason, common in the 1990s, are still very frequent in the literature (Schmidt and Mamede 2015). Indeed, more recently, as dual-process theories have gained attention, these proposals have also gained the form of interventions such as courses on clinical reasoning and cognitive bias (Norman et al. 2017). Not surprisingly, whenever trainees’ actual diagnostic performance was evaluated, the effect of these process-oriented interventions has been null or minimal (Norman et al. 2017; Schmidt and Mamede 2015). Conversely, interventions directed towards acquisition and restructuring of disease knowledge, which seems more in line with what we know about the nature of clinical reasoning and how it develops, looked much more promising. For example, an intervention directed at increasing knowledge of features that discriminate between similar-looking diseases successfully ‘immunized’ physicians against bias in reasoning (Mamede et al. 2020).

We try here to give a brief account of interventions that have been proposed for the teaching of clinical reasoning, focusing on those that have been empirically investigated and trying to relate them with the research discussed so far. Interventions that appear promising, consistently with evidence on the knowledge structures underlying diagnostic reasoning and the role of exposure to clinical problems in the development of such structures, share two basic features: they are directed at refinement of diseases knowledge and consist of exercises with clinical cases.

The *serial-cue approach* with simulation of the hypothetico-deductive model appeared in a recent review of the literature as the most prevalent intervention proposed for the teaching of clinical reasoning (Schmidt and Mamede 2015). In this approach information of the case is disclosed step-by-step, and students required in each step to generate diagnostic hypotheses and identify which additional information is needed to arrive at a diagnostic decision. The approach has rarely been investigated. While two studies showed the approach to have no effect on students’ diagnostic accuracy relative to a control group (Windish 2000; Windish et al. 2005), a recent study showed a slight advantage of using serial-cue during a learning session over employing self-explanation (Al Rumayyan et al. 2018). Its similarity to real practice may explain the widespread use of the serial cue approach, but it has been argued that it may be overwhelming for students who do not have yet developed illness scripts to guide the search for information.

*Self-explanation* as an instructional approach for the teaching of clinical reasoning has been tested in a series of studies conducted by Chamberland and colleagues (Chamberland et al. 2013, 2015, 2011) in recent years. Basically, these studies involved a learning session, in which students diagnosed clinical cases either with self-explanation, i.e., explaining aloud how the clinical features were produced, or without self-explanation, and a one-week later test. Students who used self-explanation better diagnosed similar cases in the test than their peers who had practiced without self-explanation. Students only benefitted from self-explanation on cases with which they were less familiar and which required them to extensively use biomedical knowledge, a finding that reaffirms the value of such knowledge in diagnostic reasoning. Together with deliberate reflection (see below), self-explanation has been adopted in a longitudinal curricular program at the Sherbrooke Medical school, an experience which has been recently reported (Chamberland et al. 2020).

Instructional interventions that, differently from self-explanation, focus on clinical rather than biomedical knowledge have also been proposed. These interventions foster retrieval of previous acquired clinical knowledge and elaboration on the information at hand during practice with clinical problems. Despite the different formats they may take, these interventions share the basic idea of providing students with guidance to compare and contrast different alternative diagnoses for the problem at hand. One example is concept mapping, which has been employed in various formats (Montpetit-Tourangeau et al. 2017; Torre et al. 2019) to foster students’ clinical reasoning. One of the most investigated of this type of interventions is deliberate reflection, which presents students with clinical cases that look similar but have different diagnoses (e.g. diseases that have chest pain as chief complaint) and requests students to generate, for each case, plausible diagnoses, comparing and contrasting them in light of the case features (Mamede et al. 2019, 2012, 2014). In several studies, students who engaged in deliberate reflection during practice with clinical cases provided better diagnoses for new cases of the same (or related) diseases in future tests than students who adopted a more conventional approach such as making differential diagnosis. An intervention that used deliberate reflection to strengthening knowledge of features that discriminate between similar-looking diseases has been recently shown to increase internal medicine residents’ ability to counteract bias in diagnostic reasoning (Mamede et al. 2020).

Interleaving practice, usually referred to in medical education as ‘mixed practice’, is a requirement for the abovementioned interventions. It is only possible to compare and contrast the features of clinical problems that may look similar but have in fact different diagnoses when problems of different diseases that look alike are presented together in the same exercise. The benefits of mixed practice relative to blocked practice, which presents examples of the same diagnosis together, have been demonstrated in studies comparing students’ performance when interpreting EKG after being trained either with mixed or blocked practice (Ark et al. 2007; Hatala et al. 2003).

Decreasing processing through the use of worked examples in the teaching of clinical reasoning has been more scarcely investigated. Nevertheless, indication that this intervention deserves further attention has come from a few studies exploring the influence of using erroneous examples and different types of feedback on learning diagnostic knowledge (Kopp et al. 2008, 2009) or the benefits of studying worked examples of reflective reasoning for diagnostic competence (Ibiapina et al. 2014).

Table 2 presents an attempt to summarize the extent to which these interventions for the teaching of clinical reasoning allows for the realization of the cognitive principles discussed in the first sections of this paper.

Summing up, cognitive psychology research has provided crucial contributions to guide teaching of clinical reasoning. Many of these contributions have translated into instructional interventions that have had their effectiveness empirically evaluated, with promising results. Nevertheless, as a recent review of these interventions highlighted, the existing empirical research is still scarce considering the importance of clinical reasoning in medical education. More interventions based on the conceptualizations of learning and instruction offered by cognitive psychology and more theory-driven research are much needed.

## How often do manuscripts delineating these ideas appear in advances in health sciences education?

Twenty-five years ago, the founding editors of the journal, both cognitive psychologists, and among them the first author of this article, found it necessary to create a journal in which these new approaches to medical education would feature explicitly. To what extent did they succeed? Table 2 contains the results of a search for appropriate articles in Advances in Health Sciences Education, published between 1995 and 2020. The total number of articles published in that period was 1249.

Twenty-five percent of the manuscripts published in Advances in Health Sciences Education discussed or studied the role of cognition in medical education. One could say that the initial motivation for establishing the journal has not yet entirely been fulfilled. There is clearly still room for more research into the application of these important principles of learning, expertise development, and instruction to our field.

## The future of cognition in medical education: Cognitive science

New areas hitherto not so much explored will probably attract increasing attention within medical education development and research. We refer here to artificial intelligence and to the neurosciences, both incorporated with cognitive psychology under the heading cognitive science. We discuss two examples here. First, developments in clinical practice that have strong implications for education have brought new research demands. One of these developments is the digitalization of health care, including the incorporation of artificial intelligence (Wartman and Combs 2018). Computer-based algorithms, whether derived from expert knowledge or machine learning, are expected to dramatically improve diagnostic and prognosis decisions (Obermeyer and Emanuel 2016). However, “side effects” have long been identified. For example, “automation bias” resulting from overreliance on automation systems tends to make clinicians less prone to review their initial impressions, eventually causing errors (Bond et al. 2018; Lyell and Coiera 2017). Future research should explore how clinicians can be better prepared to incorporate these developments in their practice, aiming also at better understanding the mechanisms underlying such biases and how to make trainees less susceptible to them. Moreover, the digitalization of health care has brought changes to the clinical setting that affect what students can learn from their experiences there. Think, for example, of clinical decision support systems, often associated with electronic health records (EHR), now widely adopted (Keenan et al. 2006). Patient care has been substantially altered by the widespread presence of computers, with clinical encounters now involving the ‘provider-computer-patient triangulation’ and staff rooms changed into rows of students and residents staring at computer screens. On the one hand, EHRs can be powerful educational tools. Many of them offer instant access to online learning resources at point of care. Trainees can, for example, ‘pull’ clinical guidelines or recommendations about care management during the clinical encounter. This would allow for new knowledge to be learned in a context very similar to the one in which it would be used in the future, a basic principle to facilitate retrievability. EHRs also gives trainees the possibility to easily go back to review a case and facilitates keeping track of one’s clinical experiences (Keenan et al. 2006; Tierney et al. 2013). On the other hand, potentially adverse effects have been discussed. For example, the volume of online information may be overwhelming, and trainees’ attention may be diverted from the patient to the data-entering process. More subtly, EHRs give trainees the possibility to easily convey the raw patient data to supervisors, without being compelled to interpret findings and build a narrative out of them. Incentive for the student or resident to reflect upon the problem therefore decreases, and so does the opportunity for discussion with attending physicians (Peled et al. 2009; Wald et al. 2014). How EHRs and CDDS affect trainees learning and which specific characteristics of the system itself or of its use can be optimized to foster learning are examples of areas that are likely to call attention within cognitive science research.

A second expanding research area involves the use of neurosciences tools to get insights on the processes in the brain associated with learning and expertise development. Although the complexity and cost of some of the approaches for capturing brain activity make their use less attractive, non-invasive, lower-cost tools have emerged that seem promising. Electroencephalography (EEG) signals arising from neural activities have been used to estimate students’ learning states, including within e-learning environments (Lin and Kao 2018). For example, a device that showed to be wearable proved EEG-based technology to accurately assess mental overload while surgeons performed procedures of different levels of complexity (Morales et al. 2019). Detecting mental overload in surgeons is crucial to guide the design of training programs so that situations that may bring threats to the patient or the resident can be avoided. Near Infra-Red Spectroscopy (NIRS) is another promising tool that has recently started to be employed in medical education. By measuring the level of blood oxygenation of the prefrontal cortex, NIRS provides a cost-effective alternative to other techniques such as functional Magnetic Resonance Imaging to look at the brain while students and clinicians solve problems. For example, by using NIRS in a study which trained medical students in diagnosing chest X-ray, Rotgans et al. showed that activation of the prefrontal cortex decreases with experience with a case, supporting the idea that expertise development is associated with a pattern-recognition based reasoning mode (Rotgans et al. 2019).

Trying to predict the future is always a risky endeavor, but these two areas have great potential to draw the attention of cognitive research in the coming years. If our bet is correct, we will see the products of this attention in the anniversary issue of Advances in Health Sciences Education twenty-five years from now.
